# Overexpression, purification and preliminary X-ray diffraction analysis of the controller protein C.*Csp*231I from *Citrobacter* sp. RFL231

**DOI:** 10.1107/S1744309109028681

**Published:** 2009-08-22

**Authors:** S. D. Streeter, J. E. McGeehan, G. G. Kneale

**Affiliations:** a Biophysics Laboratories, Institute of Biomedical and Biomolecular Sciences, School of Biological Sciences, University of Portsmouth, Portsmouth PO1 2DY, England

**Keywords:** DNA-binding proteins, restriction–modification systems, transcription, gene regulation

## Abstract

Restriction–modification controller proteins play an essential role in regulating the temporal expression of restriction–modification genes. The controller protein C.*Csp*231I represents a new class of controller proteins. The gene was sublconed to allow overexpression in *Escherichia coli*. The protein was purified to homogeneity and crystallized using the hanging-drop vapour-diffusion method. The crystals diffracted to 2.0 Å resolution and belonged to space group *P*2_1_. An electrophoretic mobility-shift assay provided evidence of strong binding of C.*Csp*231I to a sequence located upstream of the *csp231IC* start codon.

## Abstract 2

The crystallization of a novel controller protein is reported and its interaction with DNA is characterized.

## 1. Introduction

Controller (C) proteins have been identified in many restriction–modification (R–M) systems and play a vital role in the temporal regulation of R–M genes. These helix–turn–helix proteins have been shown to act as regulators of both their own transcription and that of the restriction endonuclease (ENase) encoded within the same operon. In some cases, C proteins also regulate transcription of the methyltransferase (MTase; Tao *et al.*, 1991 ▶; Ives *et al.*, 1992 ▶; Rimšelienė *et al.*, 1995 ▶; Lubys *et al.*, 1999 ▶; Česnavičienė *et al.*, 2003 ▶; Semenova *et al.*, 2005 ▶; Bogdanova *et al.*, 2008 ▶).

Controller proteins have recently been categorized on the basis of ten distinct DNA-recognition motifs (Sorokin *et al.*, 2009 ▶). To date, the structures of three C proteins have been reported (McGeehan *et al.*, 2005 ▶, 2008 ▶; Sawaya *et al.*, 2005 ▶); all are highly homologous proteins with similar folds and with similar DNA-recognition sites. Other groups, such as that exemplified by C.*Csp*231I and C.*Eco*O109I, have very different recognition sites and their structures are currently unknown. Kita *et al.* (2002 ▶) have previously identified the recognition sequence of C.*Eco*O109I as a 15 bp sequence comprising two palindromic pentanucleotides separated by a non­binding pentanucleotide sequence, 5′-CTAAG(N_5_)CTTAG-3′, located 47 bp upstream of the C gene start codon. This conforms to the sequence motif identified for both C.*Csp*231I and C.*Eco*O109I by bioinformatic analysis (Sorokin *et al.*, 2009 ▶).

In the present paper, we report the expression, purification and characterization of C.*Csp*231I together with preliminary crystallization and diffraction analysis.

## 2. Materials and methods

### 2.1. Cloning and expression

The gene *csp231IC* (Genbank ID AY787793.1) encoding the putative 98-amino-acid controller protein C.*Csp*231I was synthesized and subcloned into the expression vector pET-11a by GenScript (Piscataway, New Jersey, USA). The resultant expression vector was transformed into *Escherichia coli* BL21 (DE3) Gold cells. A single colony was added to 15 ml 2×YT medium containing 100 µg ml^−1^ ampicillin and cultured overnight at 310 K while shaking at 225 rev min^−1^. A 10 ml aliquot of the starter culture was used to inoculate 1 l 2×YT medium containing 100 µg ml^−1^ ampicillin and the culture was incubated at 310 K (with shaking at 225 rev min^−1^) until the *A*_600_ reached ∼0.6, whereupon 1 m*M* isopropyl β-d-1-thiogalactopyranoside (IPTG) was added to induce protein expression. The culture was incubated with shaking for a further 3 h prior to cell harvesting by centrifugation.

### 2.2. Purification

All purifications were performed at 277 K. The harvested cells were suspended in a buffer consisting of 50 m*M* Tris–HCl pH 8.0, 100 m*M* NaCl, 5 m*M* EDTA, 3 m*M* DTT and disrupted by sonication. Following centrifugation (39 191*g*, 30 min, 277 K), the supernatant was loaded onto a 5 ml HiTrap heparin HP column (GE Healthcare) and eluted with a 0.1–1 *M* NaCl gradient. Fractions containing the target protein were pooled and dialysed against 5 l buffer *A* (50 m*M* Tris–HCl pH 8.0, 100 m*M* NaCl, 1 m*M* EDTA, 1 m*M* DTT). Following centrifugation (27 216*g*, 30 min, 277 K), the supernatant was loaded onto a 1 ml HiTrap SP HP column (GE Healthcare) and eluted with a 0.1–1 *M* NaCl gradient. Fractions containing the target protein were pooled and dialysed against buffer *A* (as above) con­taining 500 m*M* NaCl to reduce interactions between the target protein and contaminants. The dialysate was loaded onto a HiPrep 26/60 Sephacryl S-100 HR (GE Healthcare) column using buffer *A* with NaCl added to a final concentration of 500 m*M*. The purified protein was then pooled and dialysed against 5 l buffer *A* prior to concentration using a 1 ml HiTrap SP HP column (GE Healthcare) with a 0.1–1 *M* NaCl step gradient. Following a final dialysis step to reduce the NaCl concentration to 100 m*M*, the protein concentration was determined by UV spectroscopy using an extinction coefficient calculated from the amino-acid sequence of the monomer of *E*_280_ = 11 460 *M* ^−1^ cm^−1^.

### 2.3. Dynamic light scattering

Dynamic light scattering (DLS) was performed on a C.*Csp*231I sample at 1.4 mg ml^−1^ in 40 m*M* Tris–HCl pH 8.0, 100 m*M* NaCl, 1 m*M* EDTA at 293 K using a Protein Solutions DynaPro temperature-controlled microsampler. The technique provides an estimate of the particle hydrodynamic radius (*R*_h_) and solution molecular weight, as well as the polydispersity of the sample, by analysis of the autocorrelation function. For globular proteins, the value of *R*_h_ can be used to estimate the molecular mass *M*_r_ using the empirical equation
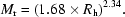

            

### 2.4. Electrophoretic mobility-shift assay

Electrophoretic mobility-shift assays (EMSA) were performed using nondenaturing gel electrophoresis. Two complementary DNA strands corresponding to the region upstream of the C.*Csp*1396I gene were purchased (Eurogentec), one of which was labelled with the fluorescent tag hexachlorofluorescein (hex), and the two strands were annealed to form a duplex. Aliquots of C.*Csp*231I were incubated with 800 n*M* hex-labelled 96 bp DNA duplex in binding buffer (50 m*M* Tris–HCl pH 8.0) at 277 K for 30 min. The samples were loaded onto a pre-run 5% native polyacrylamide gel and run at 100 V for 150 min. The gels were then scanned using an FLA-5000 imaging system (FujiFilm).

### 2.5. Crystallization

Crystallization conditions were screened by the hanging-drop vapour-diffusion method using the PACT screen kit (Molecular Dimensions) at 289 K. Drops were prepared by mixing 2 µl reservoir solution with 2 µl 1.2 mg ml^−1^ protein in dialysis buffer and were equilibrated by vapour diffusion against the reservoir solution.

### 2.6. X-ray diffraction analysis

Crystals were cryoprotected by transfer to crystallization solution containing 30%(*v*/*v*) glycerol prior to cryocooling in liquid nitrogen. Initial indexing suggested that the space group was primitive monoclinic and therefore a 180° data set was collected from a single crystal (of approximate dimensions 100 × 80 × 15 µm) with an oscillation width of 0.5° on beamline I02 of the Diamond Light Source, UK. Data extending to 2.0 Å were collected using an ADSC Q315 CCD detector and processing was performed with *MOSFLM* (Leslie, 1992 ▶) and *SCALA* (Collaborative Computational Project, Number 4, 1994 ▶). Data-collection statistics are given in Table 1 ▶.

### 2.7. Sequence analysis

Amino-acid sequence alignment was carried out using the *T-Coffee* web server (Armougom *et al.*, 2006 ▶) and visualized with the *ESPript* program (Gouet *et al.*, 1999 ▶) using the *MultAlin* similarity function with a 0.7 global score value. Secondary-structure prediction was carried out using the *ProteinPredict* server (Rost *et al.*, 2004 ▶).

## 3. Results and discussion

**Figure 1:**
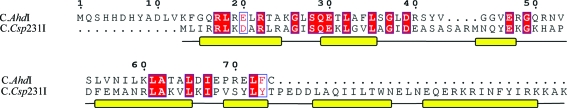
Amino-acid alignment of C.*Ahd*I and C.*Csp*231I. Identical amino acids are highlighted in red boxes and similar amino acids in white boxes. Regions predicted to be α-helical are shown as yellow bars.

Amino-acid sequence alignment of C.*Csp*231I and C.*Ahd*I revealed distinct regions of homology, particularly in the core of the protein (Fig. 1 ▶). Overall, the amino-acid sequence identity between these two proteins was 29% over 62 core residues. The main differences in the C.*Csp*231I sequence were a 12-amino-acid truncation at the N-­terminus, a four-amino-acid insertion adjacent to the predicted helix–turn–helix motif and a 32-amino-acid extension of the C-­terminal region. The *ProteinPredict* program was used to estimate the secondary structure of C.*Csp*231I. The five characteristic helices conserved among the known C-protein structures were predicted, together with two additional helices located in the extended C-­terminal region. Given the significant differences between C.*Csp*231I and the controller protein structures known to date, both in terms of protein structure and DNA-recognition sequence, we decided to embark on a structural analysis of the C.*Csp*231I protein.

**Figure 2:**
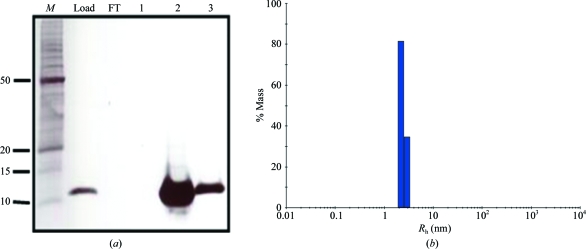
12% Tris–tricine polyacrylamide gel of purified C.*Csp*231I and dynamic light scattering of sample. (*a*) Lane *M* contains Benchmark protein ladder with selected molecular masses highlighted (kDa). Lane FT is the SP-column flowthrough. Lanes 1, 2 and 3 are selected consecutive fractions. (*b*) Single monodisperse peak corresponding to an *R*_h_ value of 2.44 nm (13.5% polydispersity).

The controller protein C.*Csp*231I was overexpressed in *E. coli* and purified to homogeneity with a final yield of 5 mg l^−1^ (Fig. 2 ▶ *a*). The molecular mass of the protein was measured as 11 360 Da by electro­spray mass spectrometry (University of Leeds, England), which is within 1 Da of that predicted from the amino-acid sequence. The hydrodynamic radius of the protein was measured as 2.4 nm by dynamic light scattering (Fig. 2 ▶ *b*), from which an estimated molecular mass of 27 kDa was obtained, suggesting that C.*Csp*231I forms homodimers in solution.

**Figure 3:**
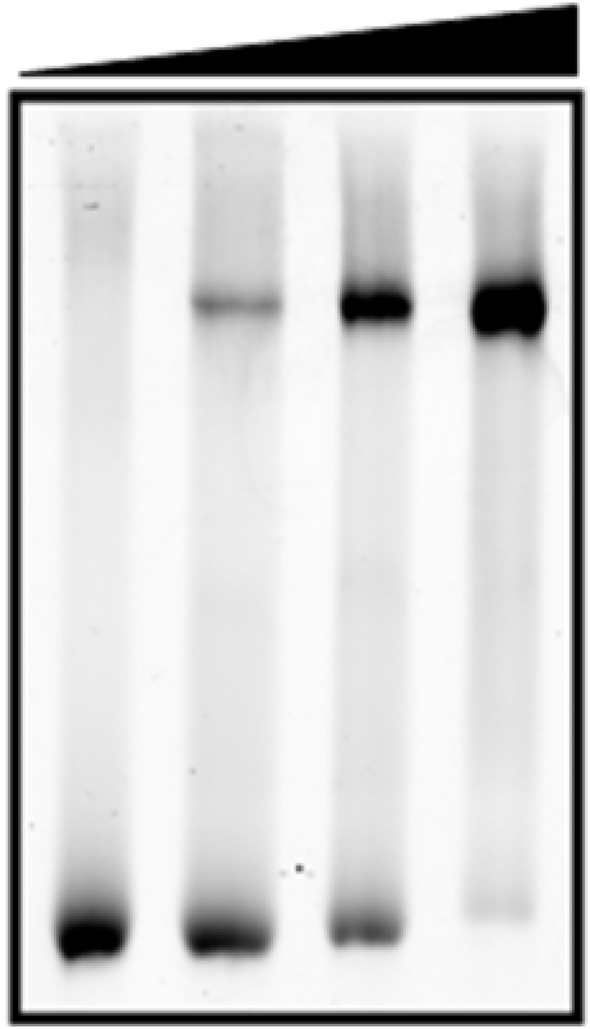
EMSA of C.*Csp*231I with hex-labelled 96 bp duplex DNA. Increasing concentrations of C.*Csp*231I were incubated with a hex-labelled 96 bp duplex DNA fragment located upstream of the *csp231IC* start codon at protein:DNA ratios of 0:1, 1:1, 2:1 and 4:1. The DNA concentration was 800 n*M* throughout.

In order to confirm the biological activity of the putative transcriptional regulator, its DNA-binding activity was assessed by an electrophoretic gel mobility assay (EMSA), using as substrate a hex-labelled 96 bp oligonucleotide corresponding to a region located directly upstream of the *csp231IC* start codon. Strong binding was observed, with a full shift at a protein:DNA ratio of 4:1 (Fig. 3 ▶), suggesting that two protein dimers may interact with this DNA sequence.

**Figure 4:**
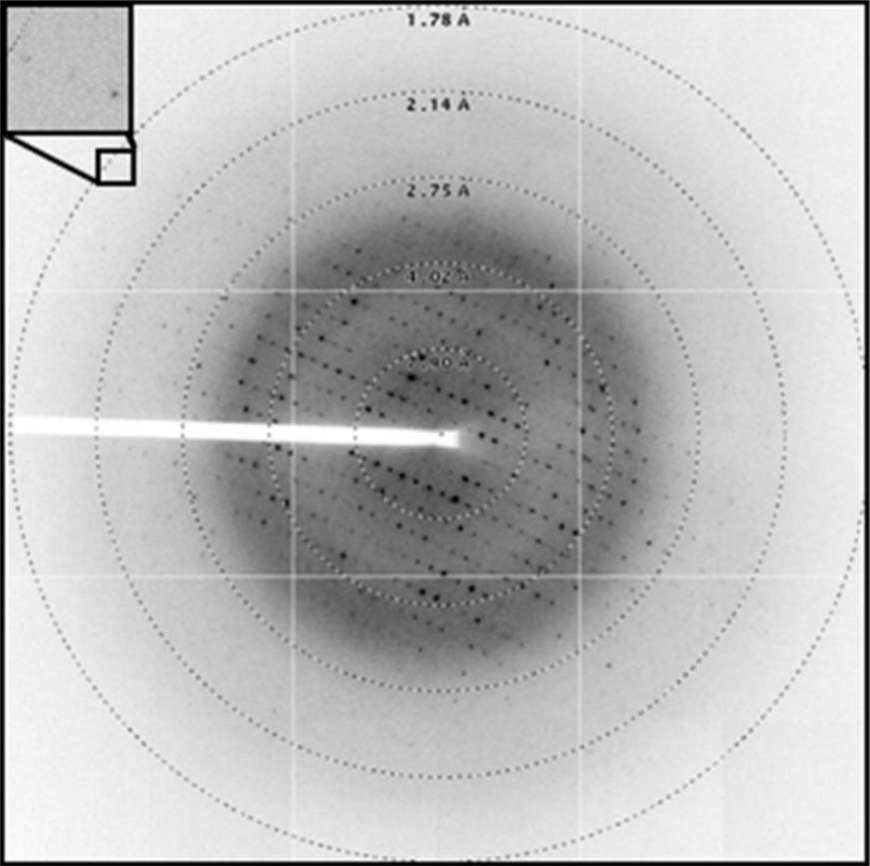
Diffraction image from a crystal of C.*Csp*231I. Reflections were observed to a resolution of approximately 1.8 Å (inset).

**Figure 5:**
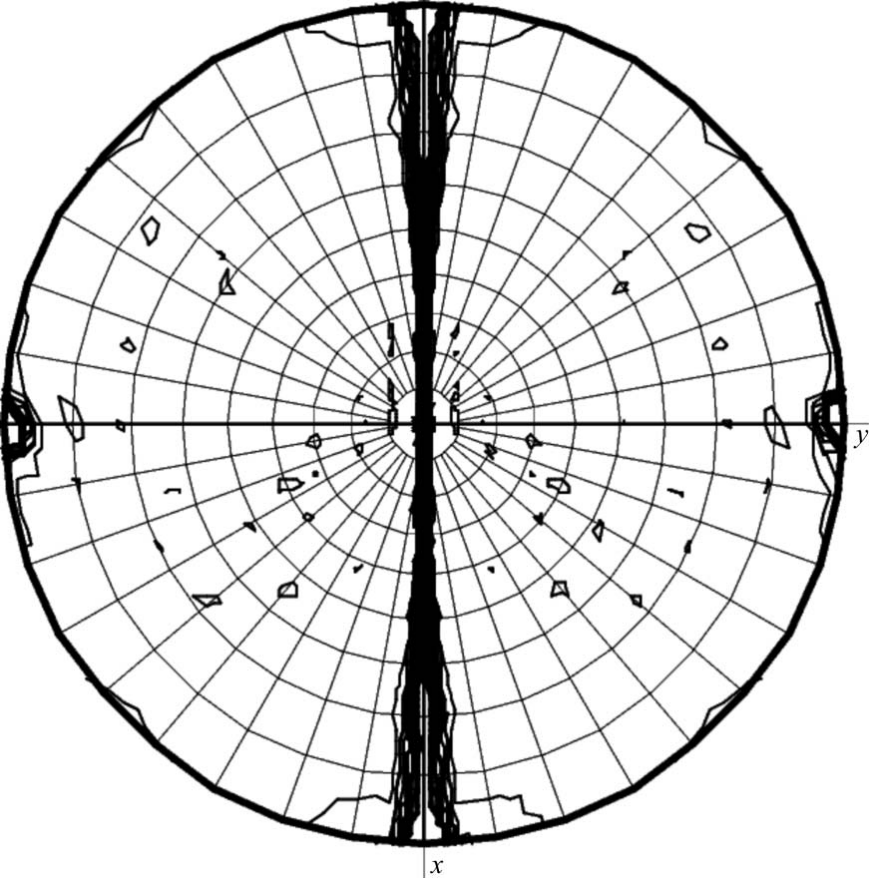
Self-rotation function showing NCS. The self-rotation function is shown using a κ angle of 180° and was calculated using data in the resolution range 20.0–4.0 Å. The radius of integration was 35 Å.

The crystallization conditions for C.*Csp*231I were obtained using the PACT screen (Molecular Dimensions Ltd) and were optimized to produce a number of single crystals suitable for X-ray analysis. Single plate-like crystals of approximately 100 µm in length were observed after one month in reservoir conditions consisting of 0.1 *M* malate–MES–Tris (MMT) pH 7.0 and 20%(*w*/*v*) polyethylene glycol (PEG) 1500. The best crystals diffracted to 1.8 Å resolution (Fig. 4 ▶), although diffraction was anisotropic and the crystals diffracted less well in other directions. Nevertheless, by probing the crystal to determine the optimum collection volume a complete data set could be collected to 2.0 Å resolution. The data were processed in space group *P*2_1_ and a self-rotation function analysis was performed using *MOLREP* (Vagin & Teplyakov, 1997 ▶). The plot at κ = 180° reveals peaks additional to those resulting from the crystallographic 2_1_ screw axis, indicating the presence of a noncrystallographic twofold-symmetry axis (Fig. 5 ▶). This suggests that C.*Csp*231I forms a dimer in the asymmetric unit of this crystal form, in common with other solved C-protein structures, resulting in a calculated Matthews coefficient of 2.01 Å^3^ Da^−1^ (Matthews, 1968 ▶).

Phase determination by molecular replacement has so far been unsuccessful. This may be an indication of significant differences in the structure of C.*Csp*231I compared with the three available search models C.*Ahd*I (McGeehan *et al.*, 2005 ▶), C.*Esp*1396I (McGeehan *et al.*, 2008 ▶) and C.*Bcl*I (Sawaya *et al.*, 2005 ▶). Indeed, such differences in structure would not be surprising given the presence of the 33-­amino-acid extension and 12-amino-acid deletion at the C- and N-­termini, respectively. Further attempts to solve the structure by molecular replacement, or if necessary by MAD, are in progress in order to provide detailed structural information on this new class of controller proteins.

## Figures and Tables

**Table 1:** Crystal parameters and data-collection statistics Values in parentheses are for the highest resolution shell.

Crystal parameters	
Space group	*P*2_1_
Unit-cell parameters	*a* = 49.01, *b* = 29.53, *c* = 64.38, α = 90.00, β = 101.91, γ = 90.00
Solvent content (%)	38.7
Data collection	
Temperature (K)	100
Wavelength (Å)	0.9795
Resolution (Å)	50.0–2.0 (2.11–2.00)
No. of measured reflections	41601 (6256)
No. of unique reflections	12439 (1821)
Completeness (%)	99.2 (99.7)
〈*I*/σ(*I*)〉	11.7 (4.1)
Multiplicity	3.3 (3.4)
*R*_merge_†	0.057 (0.276)

† *R*_merge_ = 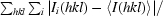 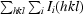, where 〈*I*(*hkl*)〉 is the mean intensity of reflection *I*(*hkl*) and *I_i_*(*hkl*) is the intensity of an individual measurement of reflection *I*(*hkl*).
